# Invertebrates, Fungal Biomass, and Leaf Breakdown in Pools and Riffles of Neotropical Streams 

**DOI:** 10.1093/jisesa/iew113

**Published:** 2017-02-28

**Authors:** Renato Tavares Martins, Lidimara Souza da Silveira, Marcos Pereira Lopes, Roberto Gama Alves

**Affiliations:** 1 Coordenação de Biodiversidade, Instituto Nacional de Pesquisas da Amazônia–INPA, Ae. André Araújo, 2936, Manaus, Amazonas CEP69067-375, Brazil (martinsrt@gmail.com); 3 Programa de Pós-Graduação em Ecologia, Instituto de Ciências Biológicas, Universidade Federal de Juiz de Fora, Juiz de Fora, Minas Gerais 36036-330, Brazil (lidimara@gmail.com); 4 Programa de Pós-Graduação em Comportamento e Biologia Animal, Instituto de Ciências Biológicas, Universidade Federal de Juiz de Fora, Juiz de Fora, Minas Gerais 36036-330, Brazil (marcosvidebula@hotmail.com; gama.alves@ufjf.edu.br)

**Keywords:** aquatic insect, ergosterol, leaf decomposition, habitat, microorganism

## Abstract

We evaluated fungal biomass (as ergosterol concentration) and invertebrate colonization during leaf breakdown of *Picramnia sellowii* Planch. (Picramniaceae) in pools and riffles of three low-order forested streams in southeastern Brazil. We hypothesized that leaf breakdown will be higher in riffles due to the high physical fragmentation and fungal activity. The experiment was carried out during the dry season of 2012, using 108 litter bags, each containing 3 ± 0.05 g of air-dried leaves. After 7, 15, 30, 60, 90, and 120 d of incubation, six litter bags (riffle = 3 and pool = 3) were removed from each stream. Leaf breakdown rate (*k*) was classified as intermediate in pools and fast in riffles. We recorded similar values of remaining leaf mass in two habitats until 60 d. However, at 90 and 120 d, this process was faster in riffles. The mean fungal biomass was similar between habitats and showed an increase during the experiment at 90 d. Fauna composition differed between habitats and across sampling dates, with Chironomidae most contributing to these differences and being particularly abundant in riffles and in the initial period of leaf breakdown (until 30 d). Shredder abundance and biomass were not different between habitats and among incubation durations. Leaf breakdown (remaining leaf mass) was positively associated with fungal and shredder biomasses. However, water velocity was not related to leaf breakdown. These findings emphasize the importance of fungal and shredder organisms, as well as the low importance of water velocity, on mass loss in low-order tropical streams.

In general, non-impacted low-order streams in tropical areas present marginal vegetation and dense canopy (Monteiro-Júnior et al. 2014, Santos et al. 2015). In these systems, due to low input of light, low primary productivity and high dependence to allochthonous matter as energy source have been recorded (Henry et al. 1994). This plant material can vary in composition, quality, and quantity between different habitats within stream (Kobayashi and Kagaya 2002) and may be used as shelter and food by aquatic invertebrates (Vannote et al. 1980, Hirabayashi and Wotton 1998). In addition, the amount of accumulated litter in each habitat may be related to physical stream characteristics; for example, the presence and abundance of obstructions (e.g., stones and fallen trees) in riffles and slow flow and low turbulence in pools (Nakajima et al. 2006).

Coarse organic matter in streams is decomposed by the action of physical, chemical, and biological factors (Gessner et al. 1999). In general, decomposers’ (principally microorganisms) activity increases at higher temperatures (beyond their tolerance level) and in well-oxygenated waters (Canhoto et al. 2013, 2016; Navarro et al. 2013.). Water velocity can act directly and positively on leaf breakdown rate due to detritus physical abrasion (Ferreira et al. 2012, Fonseca et al. 2013). Moreover, water velocity can act indirectly, stimulating fungal activity and biomass (Ferreira and Graça 2006). High current velocity (i.e., high turbulence) can stimulate sporulation by 1) reducing the time required for spore development, 2) increasing the number of conidiophores per unit area, 3) stimulating conidia detachment, or 4) facilitating nutrient acquisition (Bärlocher 1992, Ferreira and Graça 2006). Environmental factors associated with hydrology (e.g., stream velocity, width, and depth) regulate fungal composition and activity and, consequently, leaf litter decomposition in this tropical stream (Rincón and Santelloco 2009). However, it seems to be no clear pattern of fungal biomass, activity, or diversity in tropical streams, with some studies recording high values for these metrics and others reporting the opposite (Gonçalves et al. 2006, Graça et al. 2015a, Martins et al. 2015, Medeiros et al. 2015).

In addition, water velocity may determine substrate, habitat, and food distribution, influencing the structure and composition of invertebrate communities and leaf breakdown rate (Brown and Brussock 1991, Hart and Finelli 1999). For example, *Triplectides* and *Phylloicus* (Trichoptera) are the main shredders in tropical regions and are mostly recorded in pools (Nikolcheva et al. 2005). Shredders feed directly on leaf detritus and are fundamental in the leaf breakdown process (Graça 2001). In temperate streams, these invertebrates may contribute to 8–37% of leaf mass loss rates (Taylor and Chauvet 2014, Ferreira et al. 2015). However, in most Brazilian streams, shredders are scarce or absent, and microorganisms are frequently recorded as the principal decomposer acting on leaf breakdown (Gonçalves et al. 2006, 2007).

Our aim was to evaluate invertebrate colonization during leaf breakdown of *Picramnia sellowii* Planch. (Picramniaceae) in pools and riffles of tropical low-order streams. We hypothesized that leaf breakdown would be mainly influenced by physical fragmentation and fungal activity. In addition, due to the low biomass of shredders in tropical streams, we expected that these invertebrates would not increase significantly leaf breakdown. Moreover, due to their behavior and physiological needs, we predicted that invertebrate community composition and fungal biomass will be different between habitats.

## Materials and Methods

### 

#### Study Area

**Fig. 1.**
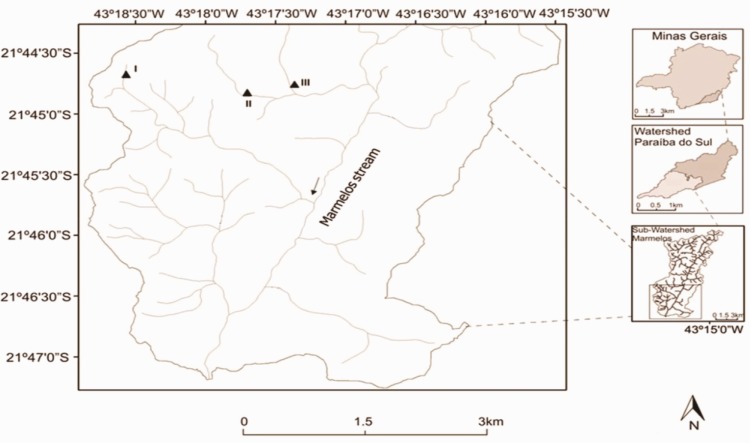
Map of the selected three low-order streams from the Ribeirão Marmelos Basin, southeastern Brazil.

We conducted the experiment in three low-order streams located in the Forest Farm, in Juiz de Fora, southeastern Brazil (Fig. 1). This farm is an area of semi-deciduous secondary forest (∼400 ha) and is contiguous with the forest of Reserva Biológica Municipal Poço D’Anta (Neto et al. 2009). The streams in this area are shallow (0.2 ± 0.1 m [SD]), narrow (1.7 ± 0.7 m), shaded by dense riparian vegetation (canopy cover = 86.11 ± 1.73%), and have near-neutral (pH = 6.7 ± 0.1) and well-oxygenated (7.9 ± 0.8 mg/L) water, with a low capacity to conduct electrical current (26.6 ± 2.9 µS/cm), and an average temperature of 17.8 ± 0.5 °C (Lopes et al. 2015). Moreover, in the bottom sand is prevailing and has 12.7 ± 1.1% of organic matter, composed principally by leaves (60.0 ± 9.1%).

#### Field Experiment

In the experiment, we used a plant species common in riparian zones of the studied streams. Green leaves of *P. sellowii* were collected and dried at air temperature. We used green leaves because they were recorded in a higher quantity in relation to senescent leaves in streams (Lopes et al. 2015). Leaves of *P. sellowii* presented 29.68 mg N/g of total biomass, 1.22 mg P/g, and 546.95 mg C/g and had low specific mass (4.9 ± 1.3 mg/cm^2^). The experiment was carried out between July and November 2012 (dry season). In each stream, 18 litter bags (20 × 20 cm, opening mesh = 1 cm) containing 3 ± 0.05 g of dry leaf were immersed into pools (*n* = 3) and riffles (*n* = 3), totaling 108 litter bags. After 7, 15, 30, 60, 90, and 120 d of incubation, we removed six litter bags (riffle= 3 and pool= 3) from each stream. Litter bags were stored in plastic sacks and transported to the laboratory in ice chests.

#### Environmental Variables

At each removal of litter bags, pH, electrical conductivity, dissolved oxygen and water temperature (Horiba model U-10, Japan), and turbidity (Lutron model TU-2016, Taiwan) were measured. At each campaign, we also estimated the water velocity by measuring the time that a plastic ﬂoat needed to move along one meter of the stream. 

#### Aquatic Invertebrates

The remaining material in each litter bag was washed under running water through a sieve with 0.21-mm mesh, sorted under a stereomicroscope, and preserved in 70% alcohol. Oligochaetes (except Megadrilli) and insects were identified at the family level according to Brinkhurst and Marchese (1989) and Merritt et al. (2008), respectively. Other invertebrates (i.e., Crustacea, Gastropoda, Hirudinea, and Turbellaria) remained at the class level. Invertebrates were classified into functional feeding groups (FFG) according to the literature (Cummins et al. 2005, Tomanova et al. 2006, Ramírez and Gutiérrez-Fonseca 2014). We did not classify Chironomidae based on the FFG due to the high number of generalist-feeding genera (Tonin et al. 2014). Crustacea were not classified into FFG due to their omnivorous behavior. In our study, Copepoda represented 90% of the sampled Crustacea. We determined only the shredders’ biomass due to the importance of these invertebrates in leaf breakdown. To determine biomass, we dried the shredders in an oven at 60ºC for 48 h and then weighed them on a precision balance (accuracy = 0.001 g; Meyer 1989).

#### Fungal Biomass

We used five leaf disks (diameter = 60 mm) to determine the fungal biomass through the concentration of ergosterol (indicative of the hyphomycete fungal biomass). These disks were frozen at −20 °C until ergosterol extraction. The extraction was carried out at 80 °C for 30 min in methanol and potassium hydroxide. The extract was purified by passing it through solid phase extraction cartridges (SPE, Waters, MA). The ergosterol retained on the column was eluted with isopropanol and quantiﬁed by high-performance liquid chromatography (Waters, MA). The mobile phase was 100% methanol, and the ﬂow rate was set to 1.4 mL/min. Final ergosterol concentrations were calculated per gram of ash-free dry mass (g AFDM) of the disks (Gessner 2005).

#### Remaining Dry Mass and AFDM

Five more leaf disks were used to determine AFDM. These disks were oven-dried at 60ºC for 72 h and weighed on a precision balance (0.001 g). Then the dried disks were placed in porcelain crucibles, which had been previously weighed on the same precision balance. The dried material was incinerated in a muffle furnace at 550ºC for 4 h and weighed again. These remaining mass is the inorganic contents (ash) present in the leaf disks. Thus, the AFDM was determined by difference between oven-dried disks mass and muffled disks mass. Final dry mass was obtained by summing the oven-dried mass of the remaining leaves and the estimated dry mass of the removed disks (n = 10).The leaf breakdown rate (*k*) was calculated according to the negative exponential model (e.g., , Petersen and Cummins 1974).

#### Data Analysis

We used a mixed-effect models, to test the differences between environmental variables, remaining leaf mass, abundance (log (*x* + 1)) and richness (*taxa* number) of invertebrates, shredders’ biomass, fungal biomass, and FFG abundance between habitats (pool and riffle), sampling periods (days) and the interaction between these two factors. We used sampling periods as a random effect to control potential temporal autocorrelation.

Nonmetric multidimensional scaling (NMDS) was used to verify the separation of invertebrate communities in relation to habitats and incubation durations. To perform this analysis, we used a Bray–Curtis similarity matrix and transformed abundance data (log (*x* + 1)). We tested the significance of differences between NMDS groups using multivariate analysis of variance (MANOVA). To perform MANOVA, we used transformed abundance data (log (*x* + 1)) and Bray–Curtis distance.

We classified leaf breakdown phases (initial or final) according to groups formed in NMDS (as described under Results; Fig. 6). Similarity percentages analysis (SIMPER; Clarke, 1993) was used to determine the percentage of dissimilarity between habitats (pools and riffles) and phases of leaf breakdown (initial and final). SIMPER was used to determine taxa that most contributed to these differences in invertebrate communities. We used the Bray–Curtis similarity matrix and nontransformed data to perform SIMPER.

We modeled the relationship between leaf breakdown (remaining leaf mass) and the main effects of independent variables using multiple regression through a Generalized Linear Model procedure (family = Gaussian). We used shredder biomass, fungal biomass, and water velocity as explanatory variables in multiple regression. Before analyzing the data to perform this analysis, we assessed the multicollinearity (*r* > 0.7) among explanatory variables by pair-wise correlation analysis (Zar 2010). All correlations between explanatory variables were <0.1. We included all variables in a multiple regression model and performed a multimodel inference analysis by backward elimination. In this analysis, we used Akaike information criterion (AIC) to select the best model. AIC allows comparing and ranking multiple competing models (Burnham and Anderson 2002). Small AIC values indicate more parsimonious models. Several models were evaluated, and we stopped simplifying the full model when the lower AIC value was recorded (Burnham and Anderson 2002). All statistical analyses were performed in program R (R Core Team 2016) using the packages “vegan” (Oksanen et al. 2013), “lme4” (Bates et al. 2015) and “lmerTest” (Kuznetsova et al. 2016).

## Results

### 

#### Environmental Variables

Water velocity was different between habitats (Table 1) and was higher (*F_1,25 _*=_* *_44.63, *P* < 0.001) in riffles (0.31 ± 0.14 m/s) than in pools (0.01 ± 0.02 m/s). However, water velocity was similar among incubation durations (*F_5,25 _*=_* *_2.02, *P* = 0.165). Water temperature (pools = 17.57 ± 1.52 °C, riffles = 17.56 ± 1.73 °C, *F_1,25 _*=_* *_0.06, *P* = 0.802), oxygen concentration (pools = 8.02 ± 1.13 mg/L, riffles = 8.24 ± 1.06 mg/L, *F_1,25 _*=_* *_0.01, *P* = 0.922), and electrical conductivity (pools = 26.99 ± 3.07 µS/cm, riffles = 26.57 ± 2.81 µS/cm, *F_1,25 _*=_* *_0.04, *P* = 0.842) were similar between habitats. However, they were different among the incubation durations (*P* < 0.010). The pH values were not different between habitats (*F_1,25 _*=_* *_1.15, *P* = 0.293) or among incubation durations (*F_5,25 _*=_* *_0.38, *P* = 0.572). The interaction between habitat and incubation duration was not significant for any environmental variable (Table 1).

**Table 1.** df, residual df, % residual deviance, and *P* of the generalized linear model testing the isolated effects of incubation duration (d) of experiments, habitat type, and their interaction on the environmental variables during leaf breakdown of *P.sellowii*

	*df*	*F*	*P*
Dissolved oxygen			
Habitats	1,25	0.01	0.922
Days	5,25	21.61	**0.010**
Habitats:days	5,25	0.65	0.428
Electrical conductivity			
Habitats	1,25	0.04	0.842
Days	5,25	8.52	**0.006**
Habitats:days	5,25	0.01	0.916
pH			
Habitats	1,25	1.15	0.293
Days	5,25	0.38	0.572
Habitats:days	5,25	1.67	0.207
Temperature			
Habitats	1,25	0.06	0.802
Days	5,25	79.69	**0.001**
Habitats:days	5,25	0.09	0.765
Water velocity			
Habitats	1,25	44.63	**<0.001**
Days	5,25	2.02	0.165
Habitats:days	5,25	1.76	0.194

*P* values: *P << 0.05 were statistically significant*

#### Leaf Breakdown

**Fig. 2.**
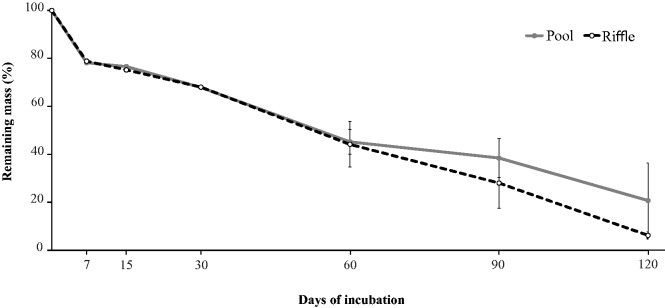
Remaining mass (mean ± SD) of leaves of *Picramnia sellowii* in pools and riffles from three low-order streams of the Ribeirão Marmelos Basin, southeastern Brazil.

The leaf breakdown rate (*k*) was 0.013 ± 0.006 d and 0.021 ± 0.001 d in pool and riffles, respectively. The percentage of the remaining leaf mass of *P. sellowii* after 7 d of experiment was 78.17 ± 0.35% in pools and 78.82 ± 0.99% in riffles (Fig. 2). At the end of the experiment (120 d), the remaining leaf mass was 24.73 ± 12.53% in pools and 6.21 ± 1.51% in riffles. The interaction between habitat and experiment duration was significant (*F_5,25 _*=_* *_6.62, *P* = 0.015; Table 2). We recorded similar values of remaining leaf mass in pools and riffles until 60 d; however, at 90 and 120 d, the leaf breakdown process was higher in riffles. The time to decompose 95% of the initial leaf litter was 230 d in pools and 142 d in riffles.

**Table 2.** df, residual df, % residual deviance, and *P* of the generalized linear model testing the isolated effects of incubation duration (d) of experiments, habitat type, and their interaction on the remaining mass, invertebrate abundance, invertebrate richness, shredder, and fungi biomasses during leaf breakdown of *P.sellowii*

	*df*	*F*	*p*
Remaining mass			
Habitats	1,25	0.60	0.442
Days	5,25	507.18	**<0.001**
Habitats:days	5,25	6.62	**0.015**
Invertebrate abundance			
Habitats	1,25	35.97	**<0.001**
Days	5,25	1.20	0.335
Habitats:days	5,25	9.45	**0.005**
Invertebrate richness			
Habitats	1,25	5.01	**0.033**
Days	5,25	0.09	0.770
Habitats:days	5,25	0.09	0.760
Shredders biomass			
Habitats	1,25	1.16	0.290
Days	5,25	0.51	0.514
Habitats:days	5,25	0.00	0.999
Fungi biomass			
Habitats	1, 21	0.88	0.359
Days	4, 21	7.89	**0.032**
Habitats:days	4, 21	1.03	0.321

*P* values: P << 0.05 were statistically significant

#### Fungal Biomass

**Fig. 3.**
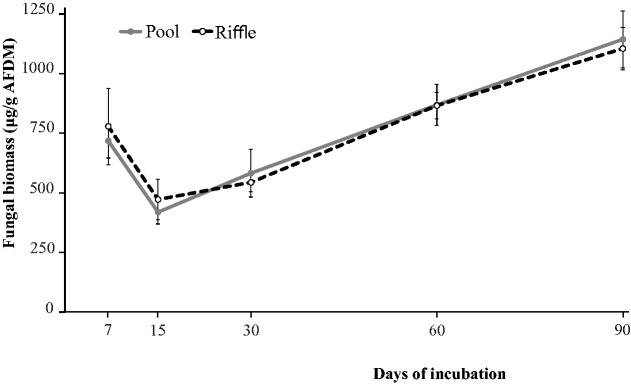
Fungal biomass (measured as ergosterol concentration; mean ± SD) during leaf breakdown of *P. sellowii* in pools and riffles from three low-order streams of the Ribeirão Marmelos Basin, southeastern Brazil.

At the beginning of the experiment (7 d), mean fungal biomass (measured as ergosterol concentration) was 717.42 ± 71.63 µg/g AFDM in pools and 778.16 ± 159.79 µg/g AFDM in riffles and was not different between habitats (*F_1,21 _*=_* *_0.88, *P* = 0.359, Table 2). The fungal biomass increased during the experiment (*F_4,21 _*=_* *_7.89, *P* = 0.032); at 90 d, the recorded mean fungal biomass was 1143.4 ± 88.3 µg/g AFDM in pools and 1104.6 µg/g ± 119.4 AFDM in riffles (Fig. 3). The interaction between habitat and experiment duration was not significant (*F_4,21 _*=_* *_1.03, *P* = 0.321; Table 2).

#### Aquatic Invertebrates

**Fig. 4.**
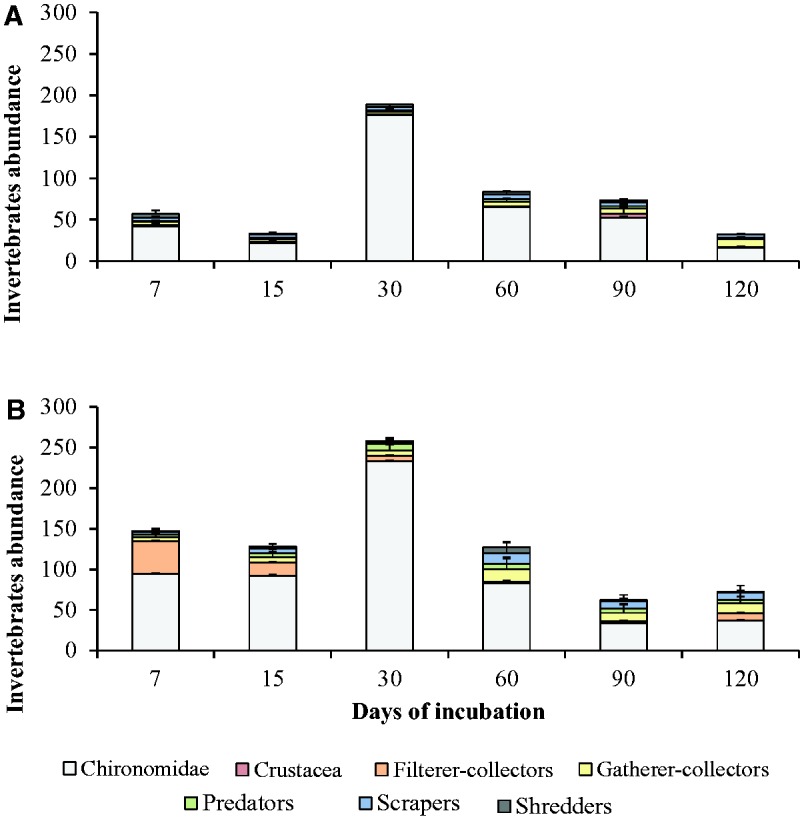
Abundance of functional feeding groups and invertebrates (sum of all FFG; mean ± SD) during leaf breakdown of *Picramnia sellowii* in pools (A) and riffles (B) from three low-order streams of the Ribeirão Marmelos Basin, southeastern Brazil.

During the experiment, we recorded 4,210 invertebrates in pools and 6,893 invertebrates in riffles. We recorded a significant interaction between habitat and experiment duration on invertebrate abundance (*F_5,25 _*=_* *_6.62, *P* = 0.015; Table 2). Higher abundance of invertebrates was observed in riffles after 7 d. of leaf breakdown process. After decreasing at 15 d, invertebrate abundance increased in both habitats at 30 d, variations being stronger in pools. Then, invertebrate abundance globally decreased in both habitats until 120 d even if a slight increase was observed in riffles between 90 and 120 d (Fig. 4).

**Fig. 5.**
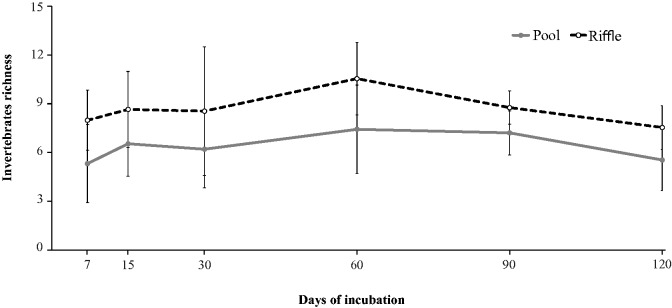
Invertebrate richness (mean ± SD) during leaf breakdown of *P. sellowii* in pools and riffles from three low-order streams of the Ribeirão Marmelos Basin, southeastern Brazil.

We recorded 38 taxa in pools and 33 taxa in riffles (Supp Table 1 [online only]). Nine *taxa* were exclusively catch in pools and four *taxa* only in riffles. The mean invertebrate richness was higher (*F_1,25 _*=_* *_5.01, *P* = 0.033) in riffles (8.68 ± 2.26) than in pools (6.39 ± 1.99; Fig. 5, Table 2). Invertebrate richness varied from 5.33 ± 2.41 taxa at 7 d to 7.44 ± 2.71 taxa at 60 d in riffles and from 7.55 ± 1.35 taxa at 120 d to 10.55 ± 2.22 taxa at 60 d in pools. However, significant differences among incubation durations were not found (*F_5,25 _*=_* *_0.09, *P* = 0.770; Table 2).

**Fig. 6.**
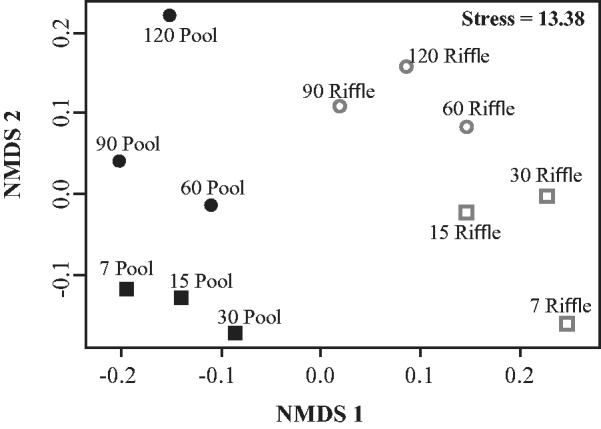
NMDS based on invertebrate abundances during leaf breakdown of *P. sellowii* in pools and riffles from three low-order streams of the Ribeirão Marmelos Basin, southeastern Brazil.

According to NMDS, it was possible to separate pools from riffles along the first axis (Fig. 6) and to separate the leaf litter bag invertebrate assemblage of the shorter (7, 15, and 30 d = initial phase) from the longer (60, 90, and 120 d = final phase) incubation periods along the second axis. The composition of faunal assemblages was different between habitats (MANOVA: *F_1,11 _*=_* *_6.97, *P* = 0.001) and among leaf breakdown periods (MANOVA: *F_1,11 _*=_* *_4.04, *P* = 0.001). The dissimilarity of pools and riffles was higher in the initial (SIMPER, 53.00%) than in the final phase (SIMPER, 38.68%; Table 3). The dissimilarity between the initial and final phases was similar in pools (SIMPER, 44.69%) and riffles (SIMPER, 47.72%). Chironomidae was the taxon that most contributed to the dissimilarities between habitats and among leaf breakdown phases (Table 3). Simuliidae mainly contributed to separate riffles from pools and was important for differentiating the initial and final phases in riffles. In the leaf breakdown final phase, habitat invertebrate communities mainly differed in abundances of Elmidae, Leptohyphidae, Hydropsychidae and Leptophlebiidae (Table 3).

**Table 3.** Mean abundance (SD) and SIMPER results indicating the contribution of taxa to dissimilarity between riffles and pools, and between initial (7, 15, and 30 d) and final (60, 90, and 120 d) phases

Taxa	Average abundance		Contribution to dissimilarity (%)
*Initial*	*Pools*	*Riffles*	**53.00**
Chironomidae	80.30 (37.33)	139.30 (81.72)	69.8
Simuliidae	0.67 (1.88)	19.78 (27.44)	16.0
*Final*	*Pools*	*Riffles*	**38.68**
Chironomidae	45.81 (51.98)	52.04 (44.85)	48.1
Simuliidae	0.00 (0.00)	4.00 (7.41)	7.8
Leptophlebiidae	4.26 (4.93)	3.67 (4.54)	4.9
Elmidae	2.08 (2.45)	4.74 (4.98)	4.8
Leptohyphidae	1.18 (1.87)	3.59 (4.34)	3.9
Hydropsychidae	0.22 (0.55)	2.15 (5.36)	3.5
*Pools*	*Initial*	*Final*	**44.69**
Chironomidae	80.30 (37.33)	45.81 (51.98)	72.3
*Riffles*	*Initial*	*Final*	**47.72**
Chironomidae	139.30 (81.72)	52.04 (44.85)	64.5
Simuliidae	19.78 (27.44)	4.00 (7.41)	15.1

In bold, values of dissimilarity (%) between groups.

#### Functional Feeding Groups

Chironomidae was the most abundant taxon in the entire experiment (Fig. 4), and its abundance was influenced by the interaction between habitat and incubation duration (*F_5,25 _*=_* *_7.02, *P* = 0.013; Table 4). The abundance of filterer-collectors (*F_1,25 _*=_* *_10.50, *P* = 0.003) and predators (*F_1,25 _*=_* *_8.48, *P* = 0.007) was higher in riffles than in pools. However, there were similar among incubation durations (*P *> 0.199). Gatherer-collector abundance was different among incubation durations (*F_5,25 _*=_* *_3.29, *P* = 0.080) and significantly higher in riffles than in pools (*F_1,25 _*=_* *_16.48, *P* = 0.015). The abundance of scrapers was near-significantly influenced by the interaction between habitat and incubation duration (*F_5,25 _*=_* *_3.69, *P* = 0.065). In pools, the scraper abundance increased until 60 d before decreasing. In riffles, scraper abundance decreased at 15 d and exhibited a strong increase at 30 d. The abundance of Crustacea (habitats: *F_1,25 _*=_* *_0.02, *P* = 0.898; incubation durations: *F_5,25 _*=_* *_1.36, *P* = 0.252) and shredders (habitats: *F_1,25 _*=_* *_0.37, *P* = 0.550; incubation durations: *F_5,25 _*=_* *_0.35, *P* = 0.587) were not different between habitats and among incubation durations (Table 4).

**Table 4.** df, residual df, % residual deviance, and *P* of the generalized linear model testing the isolated effects of incubation duration (d) of experiments, habitat type, and their interaction on the abundances of functional feeding groups, Chironomidae and Crustacea during leaf breakdown of *P. sellowii*

	*df*	*F*	*P*
Filterer-collectors			
Habitats	1,25	10.50	**0.003**
Days	5,25	2.36	0.199
Habitats:days	5,25	3.32	0.079
Gatherer-collectors			
Habitats	1,25	3.29	0.080
Days	5,25	16.48	**0.015**
Habitats:days	5,25	0.07	0.796
Predators			
Habitats	1,25	8.48	**0.007**
Days	5,25	0.00	0.988
Habitats:days	5,25	0.08	0.781
Scrapers			
Habitats	1,25	0.00	0.970
Days	5,25	1.71	0.261
Habitats:days	5,25	3.69	0.065
Shredders			
Habitats	1,25	0.37	0.550
Days	5,25	0.35	0.587
Habitats:days	5,25	0.69	0.412
Chironomidae			
Habitats	1,25	19.26	**< 0.001**
Days	5,25	1.02	0.369
Habitats:days	5,25	7.02	**0.013**
Crustacea			
Habitats	1,25	0.02	0.898
Days	5,25	1.36	0.252
Habitats:days	5,25	1.18	0.286

*P* values: P << 0.05 were statistically significant

**Fig. 7.**
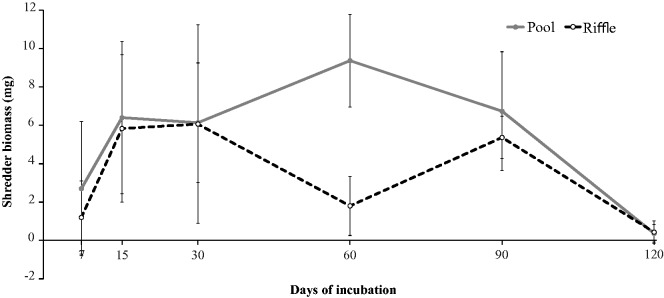
Shredder biomass (mean ± SD) during leaf breakdown of *P. sellowii* in pools and riffles from three low-order streams of the Ribeirão Marmelos Basin, southeastern Brazil.

At 7 days, the mean shredder biomass was 2.70 ± 3.50 mg in pools and 1.20 ± 1.91 mg in riffles (Fig. 7). At 120 d, the mean shredder biomass was 0.37 ± 0.46 mg in pools and 0.43 ± 0.58 mg in riffles. The interaction between habitat and experiment duration was not significant (*F_5,25 _*=_* *_0.00, *P* = 0.999; Table 2). Moreover, shredder biomass was not different between habitats (*F_1,25 _*=_* *_1.16, *P* = 0.290) and over time (*F_5,25 _*=_* *_0.51, *P* = 0.514; Table 2).

#### Multiple Regression Model

The selected multiple regression model explained 60.43% (pseudo *R*^2^) of the variation in leaf breakdown of *P. sellowii*. Leaf breakdown was positively related to shredder and fungal biomasses (Table 5). However, leaf breakdown was not related to stream water velocity.

**Table 5.** Results of the multiple regression modeling the remaining leaf mass of *P. sellowii* from three low-order streams of the Ribeirão Marmelos Basin, southeastern Brazil

	Coefficients	*t* value	*P* value
Intercept	−1.07^+1^	−1.36	0.184
Fungal biomass	5.74^−2^	6.56	**<0.001**
Shredders biomass	1.48^+3^	2.39	**0.024**

*P* values: P << 0.05 were statistically significant

## Discussion

### 

#### Leaf Breakdown

The *k* value recorded for *P. sellowii* was intermediate (0.004 < *k *< 0.017 d) in pools and fast (*k *> 0.017 d) in riffles according to Gonçalves et al. (2013), who proposed an index based on leaf breakdown rate in tropical systems. In this study, the *k* values (pools: *k* = 0.013 d; riffles: *k* = 0.021 d) were low in comparison to recorded *k* values for leaf breakdown of high nutritional quality from the Atlantic Rain Forest: *Piper divaricatum* (Piperaceae; *k* = 0.182 d), *Campomanesia xanthocarpa* (Myrtaceae; *k* = 0.052 d), *Myrcia rostrata* (Myrtaceae; *k* = 0.042 d), and *Cupania vernalis* (Sapindaceae; *k* = 0.037 d; see Moulton and Magalhães 2003, Tonin et al. 2014). Moreover, in this same Brazilian biome, our *k* values was similar to those of *Erythrina verna* (Fabaceae; *k* = 0.0202 d), *M. rostrata* (Myrtaceae; *k* = 0.019 d) and leaf mixtures from *Alchornea glandulosa* and *Cabralea canjerana* (Euphorbiaceae and Meliaceae; *k* = 0.028 d; see Moulton et al. 2010, Gonçalves et al. 2012b, Oliveira et al. 2014). These species have low hardness and high values of N and P compared to plants of other biomes, such as the Brazilian cerrado. On the other hand, *k* values of *P. sellowii* were higher than those recorded for leaves from the Brazilian Cerrado (*k* values varying from 0.009 d in *Ocotea* sp*.* (Lauraceae) to 0.001 d in *Baccharis dracunculifolia* (Asteraceae)), which are characterized by low nutritional quality (e.g.,, low % of nitrogen and phosphorus) and a high percentage of chemical inhibitors (e.g.,, tannins and polyphenols; see Moretti et al. 2007, Gonçalves et al. 2012a, Alvim et al. 2014).

In contrast to our initial hypothesis and to results reported in the literature (e.g.,, Spänhoff et al. 2007, Fonseca et al. 2013, Frainer et al. 2014, Rezende et al. 2014), water velocity did not affect the mass loss in the present work. This result may be related to low water velocity recorded in the studied streams. According to Ferreira et al. (2006), water velocity values up to 2.35 m/s had no significant effect on leaf breakdown of *Alnus glutinosa*. Moreover, leaf breakdown rate could be influenced by interactions between substrate and flow and may result sometimes in higher leaf breakdown in pools than in riffles (Hoover et al. 2006).

In our study, leaf breakdown was mainly associated with fungal biomass increase during the experiment. In tropical streams, a high decomposition rate has been related to high fungal activity (Mathuriau and Chauvet 2002). In contrary to our hypothesis and results recorded in most of the studies in tropical streams (e.g., Gonçalves et al. 2006, 2012b), shredders (even in low abundance) were important in leaf breakdown of *P. sellowii.* Tonin et al. (2014) and Martins et al. (2015) recorded a positive relationship between leaf breakdown and shredder biomass. Thus, our study reinforces the importance of measuring shredder biomass and not only shredder abundance in litter decomposition studies.

#### Fungal Biomass

We recorded rapid colonization and high fungal biomass in leaves of *P. sellowii* in both habitats, indicating good nutritional quality (e.g.,, N and P) of leaf detritus. In general, low biomass and/or abundance of fungi in detritus of low nutritional quality is expected (Graça et al. 2015b). High level of fungal colonization suggests good in-stream environmental conditions (e.g., water temperature, pH, and oxygen) for the development of these microorganisms. Sales et al. (2014) highlighted a relationship between low aquatic hyphomycete fungal biomass or sporulation and low pH and nitrogen, phosphorus, and oxygen concentrations in a Brazilian Cerrado stream. We recorded a higher fungal biomass than in other Brazilian streams (Atlantic forest: 150–274 µg^−1^ AFDM, Amazon: 374–392 µg^−1^ AFDM, Cerrado: 106–908 µg^−1^ AFDM; see Gonçalves et al. 2006, 2007, 2012; Alvim et al. 2014, Martins et al. 2015).

The increase in fungal biomass during leaf breakdown process has corroborated the results of previous studies, demonstrating a higher fungal biomass in the final period of decomposition (Gulis and Suberkropp 2003; Nikolcheva and Bärlocher 2005), probably related to increasing quality of detritus due to the leaching of chemical compounds during leaf litter decomposition. The chemical composition of detritus (e.g.,, polyphenols and tannins) and leaf toughness can inhibit colonization and increase fungal biomass (Gonçalves et al 2007). This increase may be also related to increasing nutrient availability during leaf breakdown process (Findlay et al 2002; Gessner and Van Ryckegem 2003).

In contrary to predictions, fungal biomass was similar in both habitats. An increase in fungal activity, reproduction and colonization were often recorded with increasing water velocity, mainly due to water turbulence and high oxygen concentration (Medeiros et al 2009; Gonçalves et al 2013). The similar high concentrations in dissolved oxygen in pools and riffles may have been an important factor for explaining the lack of water velocity effect on fungal biomass between habitats.

#### Aquatic Invertebrates

Invertebrate assemblages were different in pools and riffles. The higher abundance and richness in riffles, as already recorded by Buss et al. (2004) and Crisci-Bispo et al. (2007), may be related to differences in invertebrate feeding strategies. In general, invertebrate communities may be affected by amount of leaf detritus in the habitat (Egglishaw 1969), principally in riffles, where leaves are large and relatively young with low organic matter adhered. Thus, invertebrate abundance and richness may be related to continuous food flow and oxygen provided by water current to invertebrates (McCulloch 1986, Oliveira and Nessimian 2010).

Invertebrate abundance differed all along the leaf breakdown process. Higher abundance at 30 d may be related to an increase in detritus heterogeneity and quality, mostly due to microbial action and physical abrasion (Capello et al. 2004). Generally, an increase in invertebrate abundance due to degradative ecological succession is expected (Martins et al. 2011, Silveira et al. 2013, Rezende et al. 2014). However, the decrease in invertebrate abundance observed at the end of the leaf breakdown process (90 and 120 d) can be explained by an increase in support material (cellulose and lignin), due to the consumption of soft tissue and to the lower size heterogeneity of detritus (Capello et al. 2004, Leite-Rossi et al. 2016).

Higher abundance of Chironomidae larvae was recorded in riffles and in the initial phase (7, 15, and 30 d). However, this generalist, omnivorous family was abundant throughout the experiment, even in tropical species leaves (Wantzen et al. 2006). Chironomidae are less influenced by hydrological conditions than other invertebrates and may be recorded in high abundance in both habitats during leaf breakdown studies (Gonçalves et al. 2006, Moretti et al. 2007, Ligeiro et al. 2010). In contrast, Simuliidae and Hydropsychidae are filterer-collectors and favored in riffles due to the high water velocity allowing high availability of FPOM (Hamada et al. 2014). Leptophlebiidae was the most abundant *taxon* in pools. The high abundance of this mayfly family was probably related to its good adaptation to pool habitat conditions (Bonada et al. 2006; Hamada et al. 2014).

Higher abundances of filterer-collectors, gatherer-collectors, predators, and scrapers was observed in riffles. High abundance of filterer-collectors is expected in high water velocity areas due to suspended FPOM filtration needs (Brooks and Haeusler 2016). They have specialized structures (e.g.,, prologs in Simuliidae) adapted to withstand high water velocity (Allan 1995). In contrast, high abundance of gatherer-collectors has been generally recorded in pools, mainly in high FPOM deposition areas (Lemly and Hilderbrand 2000, Kobayashi and Kagaya 2002, Oliveira and Nessimian 2010). However, leaves in litter bags can trap drifting FPOM, facilitating gatherer-collector colonization (Mathuriau and Chauvet 2002). Scrapers have high affinity for riffles (Ramírez and Pringle 1998, Burk and Kennedy 2013), due to higher biofilm growth on detritus, and then higher food availability (Allan 1995, Moretti et al. 2007). High predator abundance in riffles was probably associated with higher prey availability in this habitat.

High abundance of gatherer-collectors during the final phase (60, 90, and 120 d) could be related to increasing food availability during the leaf breakdown process (Wantzen and Wagner 2006). This increase could be due to decomposers’ action (principally microorganisms) on detritus, resulting in higher FPOM availability. In addition, increasing abundances of invertebrates and microorganisms, all along the leaf litter degradation process, increase food availability for predators and scrapers (Ligeiro et al. 2010). High abundance of filterer-collectors during the initial phase (until 30 d) of leaf breakdown may be related to the only use of detritus as substrate by these organisms (Mathuriau and Chauvet 2002), and their high capacity for rapid colonization of new environments (Silveira et al. 2006). Thus, filterer-collector abundance was not related to an increase in FPOM availability.

In contrary to our hypothesis, we do not record differences in abundance and biomass of shredders between habitats and among incubation durations. The main shredders of tropical regions (*Phylloicus* and *Triplectides*) have been recorded in both riffles and pools, but with higher densities in pools (Prather 2003, Landeiro et al. 2010). In general, an increase of shredder abundance is recorded in the final period due to an increase in the biomass of microorganisms, especially fungi (Robinson et al. 1998, Gonçalves et al. 2006). Although fungal biomass increased throughout the leaf breakdown process, its values was high from the beginning of the experiment (7 d = ∼750 µg/g AFDM). Thus, fungal biomass associated with detritus was not a limiting factor for the occurrence of shredders at the beginning of the experiment. Moreover, in Amazon streams, *Mabea speciosa* leaves were colonized by shredders during the first day of the leaf breakdown process (Landeiro et al. 2008).

In conclusion, leaf mass loss was influenced primarily by hyphomycetes and by shredders. The composition of leaf litter invertebrate assemblages was influenced by habitat characteristics and the degradation process dynamics. Finally, our results have underlined the importance of 1) allochtonous leaf material from the riparian zone as a source of energy for stream invertebrates and 2) preserving riparian vegetation. 

## Supplementary Data

Supplementary data are available at *Journal of Insect Science* online.

## Supplementary Material

Supplementary Data
