# Supplementary material for: Invertebrates, Fungal Biomass, and Leaf Breakdown in Pools and Riffles of Neotropical Streams
Source: J Insect Sci. 2017 Feb 28;17(1):23. doi: 10.1093/jisesa/iew113 (PMC5388313; doi:10.1093/jisesa/iew113)
Supplement: Supplementary Data [file iew113_Supp.pdf]

Supp. Table S1. *Invertebrate abundances (mean ± standard deviation) during leaf breakdown of Picramnia sellowii in pools and riffles from three low-order streams of the Ribeirão Marmelos Basin, southeastern Brazil. FFG = functional feeding groups; FC = Filter-collectors; GC = Gatherer-collectors; P = Predators; SC = Scrapers; SH = Shredders;* = not classified in to FFG; ** = All the* Leptoceridae *catch during the study belonged to the shredding genus* Triplectides.

Taxa	FFG	Pools					
		7 d.	15 d.	30 d.	60 d.	90 d.	120 d.
COLEOPTERA							
Elmidae	GC/SC	0.33 ± 1.00	1.00 ± 1.32	0.78 ± 1.39	1.56 ± 1.74	1.78 ± 2.05	2.89 ± 3.55
Ptilodactylidae	SH	0	0	0	0	0.11 ± 0.33	0
DIPTERA							
Ceratopogonidae	P	0.11 ± 0.33	0.22 ± 0.44	0.11 ± 0.33	0.78 ± 1.72	1.00 ± 1.73	0.22 ± 0.44
Chironomidae*	-	41.89 ± 35.08	21.44 ± 14.78	177.56 ± 62.13	63.78 ± 57.95	56.44 ± 80.86	17.22 ± 17.14
Dixidae	GC	0	0	0	0	0	0
Empididae	P	0	0	0	0.11 ± 0.33	0.22 ± 0.44	0
Simuliidae	FC	0.67 ± 2.00	1.33 ± 3.64	0	0	0	0
Tipulidae	P	0	0	0	0	0	0.11 ± 0.33
Stratiomyidae	GC	0	0	0.11 ± 0.33	0	0	0.22 ± 0.44
EPHEMEROPTERA							
Baetidae	GC/SC	1.67 ± 3.08	0.89 ± 2.32	0.11 ± 0.33	0	0.11 ± 0.33	0.22 ± 0.44
Caenidae	GC/SC	0.11 ± 0.33	0	0	0	0.89 ± 2.32	0
Leptohyphidae	GC	0.44 ± 0.73	0.78 ± 1.39	0.78 ± 1.39	0.44 ± 1.01	0.33 ± 0.71	2.78 ± 3.90
Leptophlebiidae	GC/SC	4.00 ± 4.06	1.89 ± 2.37	2.22 ± 2.95	5.78 ± 5.12	3.78 ± 4.92	3.22 ± 4.76
ODONATA							

Taxa	FFG	Pools					
		7 d.	15 d.	30 d.	60 d.	90 d.	120 d.
Aeshnidae	P	0.33 ± 0.71	0	0.11 ± 0.33	0	0	0
Calopterygidae	P	0.11 ± 0.33	0.22 ± 0.44	0	0	0	0
Corduliidae	P	0	0	0	0	0.11 ± 0.33	0
Gomphidae	P	0.11 ± 0.33	0.11 ± 0.33	0	0.11 ± 0.33	0.11 ± 0.33	0
Megapodagrionidae	P	0	0.33 ± 1.00	0.67 ± 1.00	1.44 ± 1.33	1.00 ± 1.12	0
Perilestidae	P	0.11 ± 0.33	0	0.22 ± 0.44	0	0	0
Protoneuridae	P	0	0	0	0	0	0
MEGALOPTERA							
Corydalidae	P	0	0	0	0	0	0.11 ± 0.33
PLECOPTERA							
Gripopterygidae	GC/SC	0.11 ± 0.33	0	0.33 ± 0.71	0.56 ± 0.88	0.22 ± 0.44	0.22 ± 0.67
Perlidae	P	0	0	0	0.22 ± 0.44	0	0.22 ± 0.44
TRICHOPTERA							
Calamoceratidae	SH	0.56 ± 1.01	1.33 ± 1.73	1.11 ± 1.05	1.78 ± 2.49	1.33 ± 1.94	0
Glossosomatidae	SC	0	0	0	0	0	0
Helicopsychidae	SC	0.22 ± 0.67	0.44 ± 1.01	0.67 ± 0.87	1.56 ± 2.60	0.56 ± 1.01	0.22 ± 0.44
Hydrobiosidae	P	0.22 ± 0.44	0	0	0	0	0
Hydropsychidae	FC/P	0.22 ± 0.67	0.22 ± 0.67	0	0.11 ± 0.33	0	0.56 ± 1.33
Hydroptilidae	GC/SC/P	0	0	0.33 ± 0.71	0.33 ± 1.00	0	0
Leptoceridae**	SH	1.11 ± 1.54	1.11 ± 1.17	1.44 ± 1.51	1.11 ± 1.54	0.78 ± 1.99	0.56 ± 1.01
Odontoceridae	P	0	0	0	0.33 ± 1.00	0.11 ± 0.33	0.11 ± 0.33
Polycentropodidae	FC/P	0.33 ± 0.71	0.78 ± 1.56	0.67 ± 1.41	0.56 ± 1.01	0.11 ± 0.33	0.22 ± 0.67
OLIGOCHAETA							
Aelosomatidae	GC	0	0	0	0.11 ± 0.33	0.22 ± 0.44	0
Naididae	GC	0.44 ± 1.33	0.11 ± 0.33	0	1.33 ± 2.96	2.89 ± 4.27	3.00 ± 4.61
Megadrili	GC	0	0.11 ± 0.33	0	0	0	0

Taxa	FFG	Pools					
		7 d.	15 d.	30 d.	60 d.	90 d.	120 d.
Enchytraeidae	GC	0	0	0	0.11 ± 0.33	0.11 ± 0.33	0
CRUSTACEA*	-	3.44 ± 10.33	0.33 ± 0.50	0.11 ± 0.33	0.33 ± 1.00	0.33 ± 1.00	0
GASTROPODA	SC	0	0.56 ± 0.88	1.56 ± 2.74	0.78 ± 2.33	0.78 ± 1.64	0.11 ± 0.33
HIRUDINEA	P	0	0.11 ± 0.33	0.11 ± 0.33	0.11 ± 0.33	0	0
TURBELLARIA	P	0	0	0	0	0	0

Taxa	FFG	Riffles					
		7 d.	15 d.	30 d.	60 d.	90 d.	120 d.
COLEOPTERA							
Elmidae	GC/SC	0.78 ± 0.97	1.56 ± 1.59	1.22 ± 1.20	4.22 ± 3.60	3.78 ± 3.93	6.22 ± 7.41
Ptilodactylidae	SH	0	0.11 ± 0.33	0	0	0	0
DIPTERA							
Ceratopogonidae	P	0	0.44 ± 1.01	0.11 ± 0.33	0.22 ± 0.67	1.11 ± 2.67	0.44 ± 1.01
Chironomidae*	-	94.44 ± 55.87	91.78 ± 61.37	231.67 ± 127.93	83.11 ± 52.57	33.33 ± 32.02	39.67 ± 49.96
Dixidae	GC	0.44 ± 0.73	0.22 ± 0.44	0	0	0	0
Empididae	P	0	0	0.22 ± 0.44	0.56 ± 1.33	0.11 ± 0.33	0.11 ± 0.33
Simuliidae	FC	39.11 ± 42.84	15.33 ± 28.48	4.89 ± 11.01	0.44 ± 0.73	0.89 ± 1.83	10.67 ± 19.68
Tipulidae	P	0.11 ± 0.33	0.11 ± 0.33	0	0.33 ± 0.71	0.11 ± 0.33	0
Stratiomyidae	GC	0	0	0	0	0	0
EPHEMEROPTERA							
Baetidae	GC/SC	3.67 ± 2.60	2.56 ± 3.36	1.00 ± 1.66	2.89 ± 5.73	0.56 ± 1.01	1.44 ± 3.00

Taxa	FFG	Riffles					
		7 d.	15 d.	30 d.	60 d.	90 d.	120 d.
Caenidae	GC/SC	0	0	0	0	0	0
Leptohyphidae	GC	0.33 ± 0.71	1.89 ± 5.30	5.33 ± 12.29	4.22 ± 5.14	3.44 ± 5.57	3.11 ± 2.32
Leptophlebiidae	GC/SC	1.22 ± 1.39	1.78 ± 2.91	0	7.67 ± 8.25	2.44 ± 3.68	0.89 ± 1.69
ODONATA							
Aeshnidae	P	0	0	0	0	0	0
Calopterygidae	P	1.33 ± 1.73	0.67 ± 0.87	0.33 ± 1.00	0.44 ± 1.01	0	0.33 ± 0.50
Corduliidae	P	0	0	0	0	0	0
Gomphidae	P	0	0	0	0	0	0
Megapodagrionidae	P	0	0	0.11 ± 0.33	0	0.11 ± 0.33	0.11 ± 0.33
Perilestidae	P	0	0	0	0	0	0
Protoneuridae	P	0	0.22 ± 0.67	0	0	0	0
MEGALOPTERA							
Corydalidae	P	0	0	0	0	0	0
PLECOPTERA							
Gripopterygidae	GC/SC	1.11 ± 1.36	1.00 ± 1.58	1.00 ± 1.50	0.89 ± 1.69	0.44 ± 0.73	0.11 ± 0.33
Perlidae	P	0.89 ± 1.05	1.11 ± 2.03	3.33 ± 3.43	1.33 ± 1.87	1.56 ± 2.65	1.78 ± 3.70
TRICHOPTERA							
Calamoceratidae	SH	1.00 ± 1.50	2.00 ± 2.35	0.89 ± 1.36	5.22 ± 6.42	1.00 ± 1.50	0.22 ± 0.44
Glossosomatidae	SC	0	0	0	0.33 ± 1.00	0	0
Helicopsychidae	SC	0.33 ± 0.50	1.11 ± 2.67	0.44 ± 0.53	2.00 ± 2.55	1.11 ± 1.54	1.11 ± 1.96
Hydrobiosidae	P	0	0.22 ± 0.67	0.56 ± 1.67	0.56 ± 1.67	0.33 ± 0.71	0
Hydropsychidae	FC/P	1.89 ± 1.69	3.67 ± 8.80	4.11 ± 9.01	2.11 ± 5.23	3.67 ± 9.19	0.67 ± 1.66
Hydroptilidae	GC/SC/P	0	0	0.33 ± 0.71	0.89 ± 2.32	0.11 ± 0.33	0
Leptoceridae**	SH	0	0.78 ± 0.97	0.11 ± 0.33	1.11 ± 2.26	0.89 ± 1.62	0.56 ± 1.01
Odontoceridae	P	0	0	0.33 ± 0.50	0.11 ± 0.33	0	0.11 ± 0.33
Polycentropodidae	FC/P	0	0.44 ± 0.73	0.56 ± 0.73	2.22 ± 2.91	0.22 ± 0.44	0

Taxa	FFG	Riffles					
		7 d.	15 d.	30 d.	60 d.	90 d.	120 d.
Aelosomatidae	GC	0.11 ± 0.33	0	0	0	0.11 ± 0.33	0.44 ± 1.01
Naididae	GC	0.56 ± 1.01	0.67 ± 1.66	0.22 ± 0.44	3.00 ± 7.53	2.22 ± 2.73	2.44 ± 5.53
Megadrili	GC	0	0	0	0	0	0
Enchytraeidae	GC	0	0.22 ± 0.67	0	0	0.11 ± 0.33	0
CRUSTACEA*	-	0	0.22 ± 0.67	0.33 ± 0.71	0.11 ± 0.33	0.33 ± 1.00	0
GASTROPODA	SC	0	0.33 ± 1.00	0.56 ± 1.13	1.78 ± 2.73	3.78 ± 11.33	1.44 ± 3.36
HIRUDINEA	P	0	0	0	0	0	0
TURBELLARIA	P	0	0.11 ± 0.33	0.22 ± 0.44	1.22 ± 2.44	0.44 ± 1.01	0.56 ± 1.13
